# Assessment of predictive models for chlorophyll-a concentration of a tropical lake

**DOI:** 10.1186/1471-2105-12-S13-S12

**Published:** 2011-11-30

**Authors:** Sorayya Malek, Sharifah Mumtazah Syed Ahmad, Sarinder Kaur Kashmir Singh, Pozi Milow, Aishah Salleh

**Affiliations:** 1 Institute of Biological Sciences, Faculty of Science, University of Malaya, Kuala Lumpur, Malaysia; 2 Faculty of Engineering, Universiti Putra Malaysia (UPM), Serdang, Malaysia

## Abstract

**Background:**

This study assesses four predictive ecological models; Fuzzy Logic (FL), Recurrent Artificial Neural Network (RANN), Hybrid Evolutionary Algorithm (HEA) and multiple linear regressions (MLR) to forecast chlorophyll- a concentration using limnological data from 2001 through 2004 of unstratified shallow, oligotrophic to mesotrophic tropical Putrajaya Lake (Malaysia). Performances of the models are assessed using Root Mean Square Error (RMSE), correlation coefficient (r), and Area under the Receiving Operating Characteristic (ROC) curve (AUC). Chlorophyll-a have been used to estimate algal biomass in aquatic ecosystem as it is common in most algae. Algal biomass indicates of the trophic status of a water body. Chlorophyll- a therefore, is an effective indicator for monitoring eutrophication which is a common problem of lakes and reservoirs all over the world. Assessments of these predictive models are necessary towards developing a reliable algorithm to estimate chlorophyll- a concentration for eutrophication management of tropical lakes.

**Results:**

Same data set was used for models development and the data was divided into two sets; training and testing to avoid biasness in results. FL and RANN models were developed using parameters selected through sensitivity analysis. The selected variables were water temperature, pH, dissolved oxygen, ammonia nitrogen, nitrate nitrogen and Secchi depth. Dissolved oxygen, selected through stepwise procedure, was used to develop the MLR model. HEA model used parameters selected using genetic algorithm (GA). The selected parameters were pH, Secchi depth, dissolved oxygen and nitrate nitrogen. RMSE, r, and AUC values for MLR model were (4.60, 0.5, and 0.76), FL model were (4.49, 0.6, and 0.84), RANN model were (4.28, 0.7, and 0.79) and HEA model were (4.27, 0.7, and 0.82) respectively. Performance inconsistencies between four models in terms of performance criteria in this study resulted from the methodology used in measuring the performance. RMSE is based on the level of error of prediction whereas AUC is based on binary classification task.

**Conclusions:**

Overall, HEA produced the best performance in terms of RMSE, r, and AUC values. This was followed by FL, RANN, and MLR.

## Background

Eutrophication of lakes is a common global concern in lakes and reservoir. Malaysian lakes and reservoirs are also facing the same problem, as the current status of eutrophication is indicated to be more than 60% [1,2]. The adverse effects of eutrophication amongst are deterioration of water quality for human utilization, limitation of recreational usage and depletion of dissolved oxygen below acceptable level which induces reductions in specific fish and other animal populations [3]. Eutrophication promotes algae bloom hence algal biomass can be used as a good indicator of eutrophication status in lakes and reservoir around the world [4]. Chlorophyll-a have been used to estimate algal biomass in aquatic ecosystem as it is common in most algae [5]. Various models have been developed to estimate the concentration of chlorophyll-a in temperate waters such as Artificial Neural Networks (ANN), Fuzzy Logic (FL), Hybrid Evolutionary Algorithm (HEA) and Multiple Linear Regression (MLR).

Artificial neural network (ANN) model are highly flexible function approximators that can be used to model non-linear relationship. ANN models have been successfully applied to predict chlorophyll-a concentration at temperate water bodies [6-10]. Similarly, FL models can be used to model non-linear relationships. Unlike ANN, FL models can provide insight into their own operation because the fuzzy rules provide an easily understood and common sense description of the action of the FL system. FL model has been used to model eutrophication, in lakes, reservoirs, and coastal waters [11-14]. Hybrid Evolutionary Algorithm (HEA) can be used to model non- linear relationship as well. HEA uses genetic programming (GP) to generate the structure of the rule set and genetic algorithm (GA) for parameter optimization. HEA has been successfully applied to discover complex rule sets predicting the concentration of chlorophyll-a [15-17]. MLR had been used to predict chlorophyll-a concentrations based on limited number of parameters such as phosphorus and nitrogen concentrations by [3,18,19].

Comparison of the above models had been already carried out in eutrophication studies of temperate lakes [9-11,17]. The performance criteria used to assess these models were root mean square error (RMSE) and correlation coefficient (r-value). In this study, area under the ROC curve (AUC) was used as an additional performance criterion. AUC was calculated by plotting ROC curves, which were two-dimensional graphs that visually depicted the performance and performance trade-off of a classification model [20]. ROC curves were originally designed as tools in communication theory to visually determine optimal operating points for signal discriminators [21]. ROC was used in this study as chlorophyll-a concentrations can be dichotomized. For examples, different levels of eutropihication in a lake might represent either acceptable or unacceptable water quality, or concentrations of algal in the ocean might be classified as blooms if they exceed a certain threshold [22].

The aim of this study was to assess the performance of four different models, namely RANN, FL, HEA and MLR to predict concentration of chlorophyll-a in a tropical lake. To date, there are no literatures reported that assess the performance of the four models in a single study. The advantage of using AUC as an additional performance criterion is also discussed.

## Methods

### Study site and data

**Figure 1:**
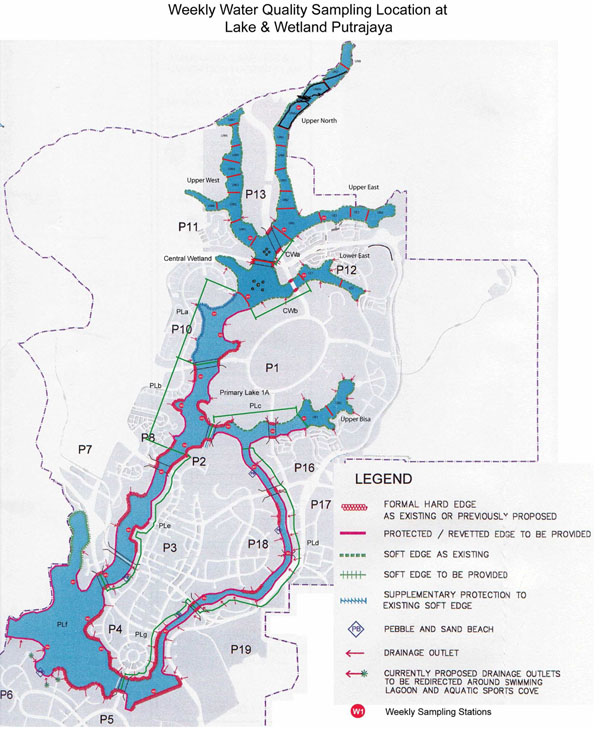
**Putrajaya Lake map.** Map of Putrajaya Lake (Malaysia) and the sampling stations for the year 2001 - 2004

Putrajaya Lake (Figure 1) which is an oligotrophic to mesotrophic man made lake covers a total surface area of more than 400 ha. It is a warm polymictic, non stratified shallow lake with an average depth of 6.6 m [23]. Algae that have been recorded from the lake are from the divisions of Bacillariophyta (11%), Chlorophyta (26%), Chrysophyta (17%), Cyanobacteria (28%) and Pyrrophyta (18%). Other characteristics of the water in Putrajaya Lake are shown in Table 1.

**Table 1:** Principal features of Putrajaya Lakes

Catchment area	Water level	Surface area	Storage volume	Average depth	Average catchment inflow	Average retention time	Circulation type
50.9 square kilometers	RL 21 meters	400 Hectares	26.5 million cubic meters	6.6 meters	200 million liters per day	132 days	warm polymictic (non stratified shallow lake)

The primary purpose of the lake is for aesthetic and for recreation, besides providing habitat for aquatic local flora and fauna. Threat from eutrophication is anticipated as due to urbanization. Hence, it is important to develop and use proper methods and techniques to monitor changes in water quality of the lake. The data used in this study was based on limnological data of Putrajaya Lake collected bi-weekly and compiled from 2001 to 2004. Water sampling procedure and sample preservation were done according to standard procedure as outlined by WHO [24] and APHA [25]. The data was arbitrary divided into three sets (sets A, B, and C). Set A was used to train and construct the models. Set B is used to the test the performance of the models. Set C is meant for cross validation of RANN model to avoid over fitting and generalization. Table 2 represents summary statistics of all variables used in this study.

**Table 2:** Summary statistics of limnological parameters from 2001 - 2004

Variables	Min	Avg	Max
Water temperature (°C)	28.42	30.29	32.33
pH	6.16	7.40	8.44
Dissolved oxygen (DO) (mg/l)	5.72	7.41	9.08
Secchi depth (m)	0.30	1.03	1.75
Turbidity (NTU)	3.50	13.68	53.10
Conductivity (Us/cm)	60.00	92.52	189.00
Ammonia nitrogen (NH3-N)(mg/l)	0.00	0.05	0.53
Nitrate nitrogen (NO3-N)(mg/l)	0.00	1.18	4.82
Chemical oxygen demand (COD)(mg/l)	2.00	18.61	79.00
Total suspended solids (TSS)(mg/l)	0.00	4.88	46.00
Chlorophyll-a (mg/l)	0.00	7.28	31.70

### Selection of input variables

In raw datasets, some variables have large variation or spread [26,27]. Normalization of the raw datasets was therefore necessary to ensure that all values of the variables are within the same range. Input data was normalized to the range 0 to 1. Less importance is given to input selection methods in many of the ecological model development [28]. Presenting large number of input to ANN, increases the network size, which leads to increase of amount of data to estimate the connection weight and possible reduction of processing speed. Similar for FL models fewer variables lead to the reduction of the dimensions for the fuzzy association matrix, and hence provide for a simpler formulation of inference rules. Preferred method of input selection should be a combination of prior knowledge and analytical approaches [29]. The analytical approach used was this study is sensitivity analysis technique for RANN and FL models. This technique measures how much a small change in one of the independent variables affects the functional value [30]. It effectively measures change in a given input affects the output across the training data set. Inputs that have large sensitivities have more importance in the mapping and therefore are the ones we should keep. The inputs with small sensitivities can be discarded. This helps the training as it reduces the size of the network, decreases the cost of data collection, and improves performance.

**Figure 2:**
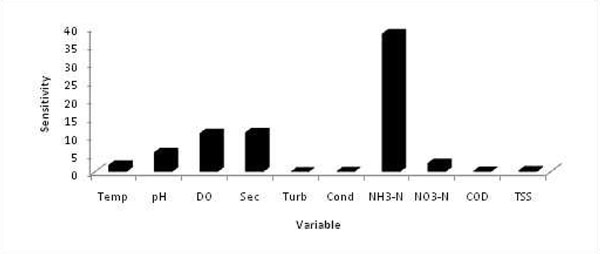
**Sensitivity Analysis Graph** Sensitivity of each input variables against chlorophyll-a concentration. Higher value on graph indicates highly sensitive variable to chlorophyll-a concentration.

Sensitivity analysis has been implemented in this study via RANN. All available variables were considered as potential input variables. Once the sensitivity level of each variable has been determined (Figure 2), backward elimination method was used to eliminate less sensitive variables and the network was retrained with reduced number of variables. This procedure was repeated, until the discarding of any extra variables did not improve the model performance. This process resulted in the inclusion of water temperature, pH, dissolved oxygen, Secchi depth, ammonia nitrogen and nitrate nitrogen for RANN and FL model development.

However for MLR model, stepwise selection was used .This was because of high correlations among the input variables were indentified. Stepwise regression is a modification of forward selection that drops variables from the model if they lose their significance as other variables are added. This has resulted in selection of only dissolve oxygen as input variable for MLR model.

HEA model optimized variable selection using general genetic algorithm (GA) approach that leads to the inclusion of nitrate nitrogen, Secchi depth, dissolved oxygen and pH. Novel crossover operator based on the non convex linear combination of multiple parents during the recombination of the population is applied for parameters optimization [15].

### Model performance comparison criteria

Three criteria for model assessment have been adopted in this study: RMSE, r and AUC. RMSE is a measure of the average level of prediction error. It indicates the absolute fit of the model to the data or how close the observed data points are to the model’s predicted values. It is shown in the following formula where y is the observed value, ỹ is the predicted value, n is the number of readings used, and j is the individual reading of the value:

The r value is a measure of correlation between the predicted and observed values of the independent variable. The r value indicates agreement between predicted and observed values but it does not indicate the performance of the models. Models are considered reliable when its predicted values correlate with observed values at r value of 0.5 or above.

In this study, AUC was calculated from ROC curve graphs. ROC curve is a graphical plot of sensitivity or true positive rate versus false positive rate. In order to plot the ROC curve the concentration of chlorophyll-a was scaled according to different eutrophication scales [31]: ultra-oligotrophic (<=1.00*μ*g/L), oligotrophic (1.00–2.50 *μ*g/L); mesotrophic (2.5–8.0*μ*g/L), eutrophic (8.0–25.0 *μ*g/L) and hypertrophic (>=25 *μ*g/L). The AUC was then calculated to determine model performance using the trapezoidal rule. Trapezoids are formed using the observed points as corners and computing the areas of these trapezoids and adding them up. Thresh-hold values of AUC is adopted from [32]. AUC ranges from 0 to 1, where a score > 0.9 indicates outstanding discrimination, a score between 0.8–0.9 is excellent, and a score > 0.7 is acceptable.

### RANN model development

In the study, RANN [33] was used for model development. It followed deterministic modelling approach where the system state at time (t) was measured by system state at time (t-1) and the copied weights of time ( t-1 ) was used as feedback input to determine weights at time t. RANN was a modification to feed-forward neural network structure. RANN network geometry was determined via trial and error. One hidden layer RANN with back-propagation through time learning was employed. It has been reported that only one hidden layer is needed to approximate any continuous function [34]. The geometry of the RANN model (using water temperature, pH, dissolved oxygen, Secchi depth, ammonia nitrogen and nitrate nitrogen data as inputs) was 6-4-l (number of inputs-number of hidden nodes-number of outputs).

Back – propagation through time (BPTT) [35] a learning algorithm for RANN network was used in this study. The Back-propagation through time (BPTT) algorithm is based on changing the network from a feedback system to feed-forward system by folding the network over time. The network uses momentum learning algorithm to determine the weights in the network. This algorithm is an improvement to the gradient-descent search, where previous increment to the weight is used to speed up and stabilize convergence. In momentum learning the equation to update the weights are as follows:

wij(n +1) = wij (n) + η∂i(n)xj(n) + α(wij (n) – wij(n-1)

Where α is the momentum constant. Weights adaptation using momentum learning is changed proportionally to how much they were updated in the last iteration. Momentum learning is a robust method to speed up learning, and it suitable as the default search rule for networks with nonlinearities [36].

The size of the steps taken in weight space during the learning phase is a function of a number of internal network parameters including the learning rate, momentum value, error function, epoch size and gain of the transfer function. Appropriate step sizes, and hence, appropriate combinations of network parameters in this study, are determined by trial and error. Change in algal biomass is known to be nonlinear process [37]. To introduce nonlinearity to system hyperbolic tangent function is used at as an activation function at the hidden layer and at the output layer.

f(x) = tanh(αx)

α is a slope parameter and is set to 0.7, learning rate of 0.01 was used . An epoch size used is 100.

Generalization ability of a network, as measured by the RMSE between the predicted and historical values of an independent test set, changes as training progresses is a function of the size of the steps taken in weight space . In order to optimize model performance, three data sets: are used for the RANN model development in this study a training set (dataset A), a test set (dataset B), and a validation set (dataset C). The test set was used to evaluate the generalization ability of the network. The validation set was used to assess the performance of the model once the training phase has been completed. The process of cross-validation removes the risk of the neural network memorizing the data [38].

### FL model development

The FL model used to model chlorophyll-a concentration was adopted from [14]. The model development constitutes three basic steps.

#### Data reduction

Variables used to develop the FL model were selected through sensitivity analysis generated from RANN. Fewer variables lead to the reduction of the dimensions for the fuzzy association matrix, and hence provide a simpler formulation of inference rules [13,39]. Water temperature, pH, dissolved oxygen, Secchi depth, ammonia nitrogen and nitrate nitrogen was used as input variables.

#### Clustering of input and output data

Self-organizing maps (SOM) [40], an unsupervised learning method, was used to cluster input and output data. SOM is common technique used to analyse multi-dimensional data [27,41] by providing for a reduced dimensional illustration. In this study, SOM was used to construct a two dimensional graph that described the clustering of input and output variables, whereby the clusters for input parameters can be mapped directly to the cluster of chlorophyll-a. In addition, the SOM is used to obtain mean values as well as 97.5% confidence interval bands of each cluster of model variables. If there was no statistically significant overlap between the clusters, the classification result is considered acceptable. The procedure of SOFM can be found in [42,43]. Clusters obtained from SOM were used to extract information for defining the fuzzy membership functions and constructing the fuzzy inference rules.

#### Defining membership functions

**Figure 3:**
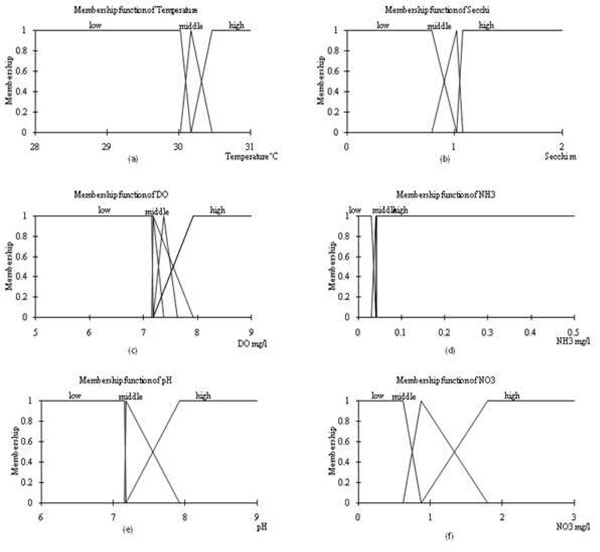
**Fuzzy membership for selected input variables.** Fuzzy membership diagram (a) water temperature, (b) Secchi depth, (c) dissolved oxygen, (d) Ammonia Nitrogen and (f) Nitrite Nitrogen

The mean value (μ) of each cluster membership was assigned to 1.0. Function type and membership function was defined for each variable. These are shown in Figure 3.

Strategies for inference rules induction, extended case reasoning has been adopted in this study as explained in [14]. A total of 64 inference rules were generated for the chlorophyll-a FL model from the training dataset A. 16 rules were extracted to determine high algae biomass, 18 rules were determined for medium algae biomass and 30 rules extracted to determine low algae biomass. In order to conduct quantitative comparison, the developed models were tested with dataset B. This was to avoid biasness in results. The outputs for FL model were defuzzified by centre of gravity method and plotted together with observations.

### HEA model development

**Figure 4:**
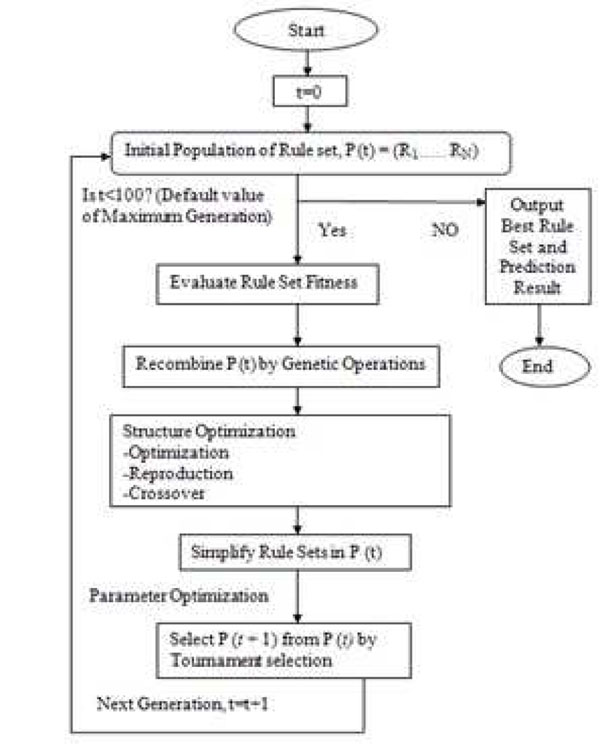
**HEA structure diagram.** The HEA structure adopted from Cao et al.[15].

Hybrid Evolutionary Algorithm (HEA) evolved from Evolutionary Algorithm (EA) with additional features of parameter optimization. The main characteristic of HEA is ability for solving problems involving complexity, noisy environment, imprecision, uncertainty and vagueness. Due to this characteristic of HEA structure (Figure 4) has been adopted in this study as explained in [15]. HEA uses GP to create and optimize the formation of rule sets and a GA to optimize the parameters of a rule set . GP is an expansion of GA where the genetic populations are represented as computer programs of varying sizes and shapes. In a typical GP, computer programs can be depicted as parse trees, and a branch node depicts an element from a function set (arithmetic operators, logic operators, elementary functions of at least one argument), and a leaf node depicts an element from a terminal set (variables, constants, and functions of no arguments). These symbolic programs are later assessed using ‘fitness cases’. Fitter programs are chosen for recombination to form the next generation by means of genetic operators, for example crossover and mutation. This process is iterated for successive generations until the termination criterion is fulfilled. A general GA was applied for parameter optimization of the random parameters in the rule set. 100 runs were conducted independently for each data set. For simplicity, we set the maximal rule size to be 1 (single rule). All the experiments were performed on a University of Malaya High Performance supercomputer (Altix SGI 1300) using the programming language C. Data from dataset A was used to generate rules. The rule generated by HEA was latter tested using dataset B which was not used for training to avoid biasness in result.

### MLR model development

In this study, MLR model was developed from dataset A using multiple regression procedure of the SPSS 17.0 software. The MLR model was developed using stepwise selection method as there exists high correlation between independent variables. Chlorophyll-a concentration was assigned as the dependent or criterion variable. The stepwise variable selection method identified dissolved oxygen as the independent variables. The developed models were tested with dataset B. This was to avoid biasness in results.

## Results

**Figure 5:**
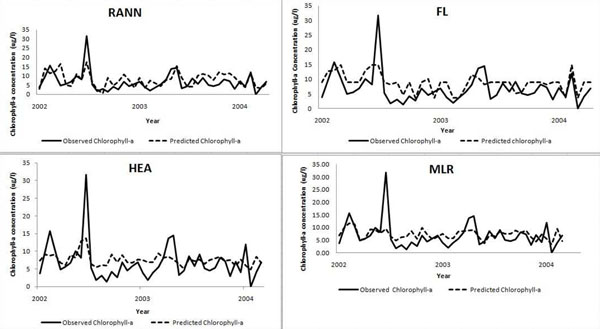
**Graph for observed verses predicted data for the prediction models.** Graph illustrates observed against predicted data using testing dataset B for the RANN model (Figure 5, top left), FL model (Figure 5, top right), HEA model (Figure 5, bottom left) and MLR model (Figure 5, bottom right).

**Figure 6:**
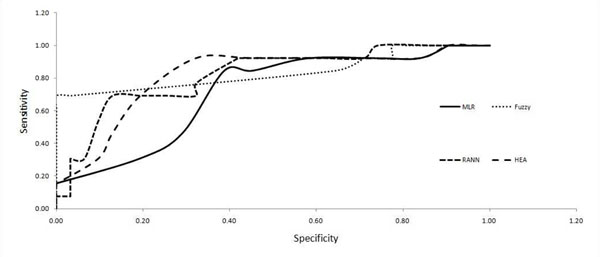
**ROC curve graph**. ROC curve plotted for sensitivity (true positive) against specificity (true negative) for varied concentration of chloroplyll-a

Graph for predicted over observed data for each model is plotted (Figure 5) using testing dataset B to avoid biasness in result. Graphical representation of the graph indicated high concentrations of chlorophyll-a (above 30 ug/L) are not predicted well by all the models. This might be related to low incidence of high concentration of chlorophyll-a in the lake. Putrajaya Lake is categorized as an oligotrophic lake with low productivity. Besides RMSE and r value (Table 3) calculated for each model for testing dataset B, AUC value was calculated as well by using ROC curves (Figure 6).

**Table 3:** Result summary for chlorophyll-a prediction models

Models	RMSE	r	AUC
MLR	4.60	0.5	0.76
FL	4.49	0.6	0.84
RANN	4.28	0.7	0.79
HEA	4.27	0.7	0.82

Chlorophyll-a prediction result for RANN, FL, HEA and MLR model based on three performance criteria RMSE, r and AUC.

HEA model was used to generate rules to discover relationship between cholorophyll-a concentrations and water quality parameters at Putrajaya Lake. The best rule set in terms of minimal testing error in 100 runs is given below.

IF ((((NO3N*126.117)>=5.273)OR(Secchi>154.513))AND(((NO3N*444.685)*(NO3N*13.361))<444.685))

THEN chlorophyll-a=(96.579/DO)

ELSE

Chlorophyll-a=(DO-ln(|((61.273/pH)-DO)|))

The MLR model equation using stepwise variable selection for Putrajaya Lake is as follows: Chlorophyll-a = -11.685 + 2.732.DO.

## Discussion

Same data set was used to assess the performances of the four models: RANN, FL, HEA and MLR. The RMSE value indicates that both HEA (4.27 ug/l) and RANN (4.28 ug/l) performed better than FL (4.49 ug/l) and MLR (4.60 ug/l). RMSE values obtained in this study were comparable with similar models developed for chlorophyll-a estimation at temperate lakes. FL model developed for temperate lakes recorded RMSE value of (7.0 ug/l) [13] and HEA model reported RMSE value of (39 – 87 ug/l) [15]. All the models are generally reliable as their predicted values correlate with observed values with r value of 0.5 or above. HEA and RANN produced similar performance when used for predicting phytoplankton biomass at temperate lakes [17]. Previous studies have also shown that RANN and FL performed better than MLR model [9-11]. This is consistent with findings of the present study.

Based on AUC rating in [32], FL (AUC value 0.84) and HEA (AUC value 0.82) can be categorized as excellent prediction models of chlorophyll-a concentration. RANN (AUC value 0.79) and MLR (AUC value 0.76) are categorized as acceptable models of chlorophyll-a concentration.

Performance inconsistencies between four models in terms of performance criteria in this study resulted from the methodology used in measuring the performance. RMSE is based on the level of error of prediction whereas AUC is based on binary classification task. Better performance of FL model over RANN and HEA might be due to collapsing continuous response (chlorophyll-a concentration) into two values. Theoretically and empirically that AUC is a better measure for model evaluation than accuracy. RMSE meanwhile measures model accuracy. Many ecological responses are difficult to measure accurately and definitely. Therefore AUC is suitable for characterizing responses that are dichotomous such as lake eutrophication [44].

Dissolved oxygen was used to predict chlorophyll-a concentrations for the MLR model. Other variables were not used because they are highly correlated. Highly correlated variables were excluded stepwise during the process of constructing the MLR model. The use of MLR model to predict chlorophyll-a has serious drawbacks as the model is oversimplified. Eutrophication is a complex process with non linear relations between environmental variables and therefore cannot be explained with simplistic approach. Sensitivity analysis to select variables as used in RANN and FL determine the contributions of the independent variables and the way they act on the dependent variable. Sensitivity analysis adds strength to ANNs in their explanatory capacity. More importance is placed on variables that have large sensitivities. Variables with small sensitivities are discarded. This is important as the effect of presenting large number of input to ANN, increases the network size, which leads to increase of amount of data to estimate the connection weight and possible reduction of processing speed. Similarly for FL model large number of input causes difficulty in defining fuzzy members. Both RANN and FL models were developed using the final selected variables such as water temperature, Secchi depth, pH, ammonia nitrogen, dissolved oxygen and nitrate nitrogen. Chlorophyll-a concentration are related to algal biomass and concentration of chlorophyll-a in this study represent the five major division of algae that is Bacillariophyta, Chlorophyta, Cynanobacteria , Chrysophyta and Pyrropytha. It is well known that temperature can enhance phytoplankton growth rate [45,46]. Cyanobacteria and Chlorophyta which comprises 28% and 26% of algae population are identified as major contributor of chlorophyll-a concentration in Putrajaya Lake. Cyanobacteria and Chlorophyta are known to prefer high water temperature [47-49]. Inability to grow at high pH is a characteristic of oligotrophic species mainly desmids which comprises of major population of algae at Putrajaya Lake [50]. It can be inferred that algae abundance at Putrajaya Lake are controlled by pH concentration. The nutrients, both ammonia nitrogen (NH3-N) and nitrate nitrogen (NO3-N) are among parameters selected by sensitivity analysis. Nutrients inputs into oligotrophic lakes often increase phytoplankton biomass and productivity [51]. Secchi depth is correlated with chlorophyll-a measurements. In many standing waters, determination of Secchi depth has been found to be a simple and reliable approach to monitoring changes in seasonal phytoplankton biomass. Meanwhile it is typical to find higher levels of oxygen in depths where larger concentrations of phytoplankton are found [52].

Even though ANN models are able to make perfect predictions and are recognised as powerful, they are considered to be ‘black-box’ in nature. Therefore explanatory methods such as FL and HEA have been adopted in this study with the idea to clarify the ‘black-box’ approach of ANNs. An FL approach proves to be a practical and successful technique when dealing with semi-qualitative knowledge and semiqualitative data [53] which is, for example, the case when trying to model algal biomass or algal blooms. However, the definition of appropriate membership functions and the induction of inference rules, common to any FL modelling approach, remain difficult, since these very much depend on specific knowledge and expertise of any particular ecologist [54]. HEA approach can overcome the limitation of FL and ANN approach. HEA allows discovery of predictive rule set in complex ecological data. The genetic algorithm used in HEA provides parameter optimization which resulted in the inclusion of nitrate nitrogen, Secchi depth, dissolved oxygen and pH for chlorophyll-a concentration estimation at Putrajaya Lake. The HEA rule sets discovered for chlorophyll-a concentrations at Putrajaya Lake is rather complex. The IF branch of the discovered rule set explains chlorophyll-a concentration can be determine by using dissolved oxygen when concentration of nitrate and Secchi depth are reported to be high. If this condition is not meet chlorophyll-a concentration is determine using the ELSE branch, where pH and dissolved oxygen is used. This can be justified by findings postulated in literature. Nutrients such as nitrates increase algae biomass. Concentrations of chlorophyll-a can be determined using dissolved oxygen as algal photosynthesis is usually the major supplier of oxygen to slow flowing water body. Dissolved oxygen and pH value in natural waters is primarily associated with photosynthesis [55].

## Conclusions

This paper presents an assessment of RANN, FL , HEA and MLR approaches in modelling chlorophyll-a of a tropical lake in Malaysia. In this study FL and HEA models produced promising results. FL and HEA approach prove to be practical and successful techniques when dealing with limited datasets of complex relationship without clear distinction of memberships. RANN model are data-driven models which is difficult to calibrate and requires a large number of datasets to perform accurate prediction. In this study FL, RANN and HEA give similar results and are potential algorithms to be deployed as water management tools as compared to MLR.

## Competing interests

The authors declare that they have no competing interests.

## Authors' contributions

SM headed the study and structured the whole research for all of the models development of and manuscript writing. SMSA assisted in FL model development and SKK assisted in MLR model development. PM , SKK, SMSA and AS assisted in manuscript writing. All authors contributed in this study.
